# Low-density hepatitis C virus infectious particles are protected from oxidation by secreted cellular proteins

**DOI:** 10.1128/mbio.01549-23

**Published:** 2023-09-06

**Authors:** Christelle Granier, Johan Toesca, Chloé Mialon, Maureen Ritter, Natalia Freitas, Bertrand Boson, Eve-Isabelle Pécheur, François-Loïc Cosset, Solène Denolly

**Affiliations:** 1 CIRI – Centre International de Recherche en Infectiologie, Univ. Lyon, Université Claude Bernard Lyon 1, Inserm, U1111, CNRS, UMR5308 ENS de Lyon, Lyon, France; 2 Centre Léon Bérard, Centre de Recherche en Cancérologie de Lyon, CNRS 5286, Inserm U1052, Université Claude Bernard Lyon 1, Lyon, France; 3 Department of Infectious Diseases, Molecular Virology, Heidelberg University, Heidelberg, Germany; Columbia University Medical Center, New York, New York, USA; University of North Carolina at Chapel Hill, Chapel Hill, North Carolina, USA

**Keywords:** hepatitis C virus, oxidation, virus stability

## Abstract

Hepatitis C virus (HCV) particles secreted from cells are stable at 37°C, whether the producer cell media contain serum or not. Yet, we found that intracellular HCV particles harvested after freeze-thawing of producer cells are highly unstable upon resuspension in a serum-free medium, indicating that either HCV particles gain intrinsic stability during their secretion and egress from producer cells or, alternatively, that a factor secreted from cells can stabilize intrinsically unstable HCV particles. We aimed at investigating either possibility and unraveling the mechanisms evolved by HCV to promote the stability of its viral particles. We showed that after purification and resuspension in a serum-free medium, HCV infectious particles released in cell supernatants are quickly and specifically degraded at 37°C in comparison to other viruses that can infect hepatic cells. We also found that cell-secreted proteins, including human serum albumin and transferrin, could protect HCV particles from this loss of infectivity. Moreover, we showed that such protection mainly impacted low-density particles (*d* < 1.08), suggesting a specific alteration of viral particles that are lipidated. Since we also demonstrated that neither HCV RNA nor surface glycoproteins were altered, this suggested that virion lipids are sensitive to decay, resulting in a loss of infectivity. Indeed, our results further indicated that HCV particles are sensitive to oxidation, which leads to a loss of their membrane fusion capacity. Altogether, our results indicate that HCV is highly sensitive to oxidation and highlight a specific protection mechanism evolved by HCV to prevent oxidation-mediated degradation of its lipidated particles by using secreted factors.

**IMPORTANCE:**

Assessments of viral stability on surfaces or in body fluids under different environmental conditions and/or temperatures are often performed, as they are key to understanding the routes and parameters of viral transmission and to providing clues on the epidemiology of infections. However, for most viruses, the mechanisms of inactivation vs stability of viral particles remain poorly defined. Although they are structurally diverse, with different compositions, sizes, and shapes, enveloped viruses are generally less stable than non-enveloped viruses, pointing out the role of envelopes themselves in virus lability. In this report, we investigated the properties of hepatitis C virus (HCV) particles with regards to their stability. We found that, compared to alternative enveloped viruses such as Dengue virus (DENV), severe acute respiratory syndrome coronavirus 2 (SARS-CoV-2), hepatitis delta virus (HDV), and Crimean–Congo hemorrhagic fever virus (CCHFV) that infect the liver, HCV particles are intrinsically labile. We determined the mechanisms that drastically alter their specific infectivity through oxidation of their lipids, and we highlighted that they are protected from lipid oxidation by secreted cellular proteins, which can protect their membrane fusion capacity and overall infectivity.

## INTRODUCTION

Viruses can be transmitted through highly divergent routes, such as direct person-to-person contacts, droplets, aerosols, or interactions with a plethora of environment-dependent factors/vectors. Assessments of viral stability on surfaces or in body fluids under different environmental conditions and/or temperatures are often performed, as they are key to understanding the routes and parameters of viral transmission and to providing clues on the epidemiology of infections. Several reports have demonstrated that the life span in different environments of coronaviruses such as severe acute respiratory syndrome coronavirus 2 (SARS-CoV-2) and Middle East Respiratory Syndrome coronavirus (MERS-CoV) is markedly higher as compared with that of other enveloped viruses, such as, e.g., influenza A virus (1). However, for most viruses, the mechanisms of inactivation vs stability of viral particles remain poorly defined. For example, although many studies have indicated that enveloped viruses are generally less stable than non-enveloped viruses (2 – 4), they are structurally highly diverse, with different compositions, sizes, and shapes, which may differentially impact their stability. Yet, the current data indicate that their envelopes themselves play a crucial role in virus lability. Indeed, while lipid bilayers of the envelopes provide an additional physical protection of the nucleocapsid from the environment, they are intrinsically fragile and can be easily altered. This may result in a loss of infectivity through disruption of the viral surface glycoproteins and hence, of the cell entry processes. For example, some retroviruses exhibit particularly labile envelopes due to easy shedding of the SU (surface) subunit from TM (transmembrane) subunit of their glycoprotein complexes, whereas rhabdoviruses such as vesicular stomatitis virus display particularly stable surface glycoproteins (5, 6).

Among enveloped viruses, viruses that infect the liver may have evolved specific mechanisms for maintaining their specific infectivity, as they face several challenges upon their assembly and release. On the one hand, they are produced in hepatocytes, i.e., professional secretory cells that are characterized by high and diverse metabolic activities resulting in secretion of thousands of proteins per second (7), and on the other hand, they are further exposed to a different and highly complex environment after their secretion. Indeed, the liver is a complex organ that plays a central role in metabolism homoeostasis (8). Specifically, it is involved in the metabolism of lipids, with, e.g., synthesis of bile and secretion of lipoproteins, as well as in the metabolism of carbohydrates with gluconeogenesis and glycogenolysis. It is also responsible for the synthesis of a large fraction of serum proteins, such as for example albumin, apolipoproteins, alpha-1-antitrypsin, or several growth factors. Many of the above-mentioned factors are secreted via the same organelles than those that are responsible for secretion of viral particles, with particularly high concentrations, which can increase their encountering and mutual interrelations.

In this report, we sought to address the properties of hepatitis C virus (HCV) particles with regards to their stability. HCV infection is a major cause of chronic liver diseases worldwide. Direct-acting antivirals can now cure most patients but there remains major challenge in basic, translational, and clinical research (9). As an enveloped virus, the HCV particle is composed of a nucleocapsid containing the viral (+) RNA and core proteins, surrounded by a lipid bilayer in which the viral E1 and E2 surface glycoproteins are embedded. It is still unclear if the HCV p7 viroporin, which plays a crucial role in envelopment of viral particles (10), can be incorporated in virions. In addition, HCV particles are associated with neutral lipids and apolipoproteins (11 – 16), which is the result of direct lipid transfer of lipids from lipoproteins and protein components of the serum during secretion or after virion egress (15, 17).

HCV particles produced in cell culture (termed HCVcc) can survive in a liquid environment for some time with a stability inversely correlated to temperature (18). While some secreted components like, e.g., hepatic lipase and lipoprotein lipase may alter HCV particles, leading to a loss of infectivity (19 – 21), the presence of human serum in tissue culture media was shown to increase the stability of these particles (18). However, it also modulates the composition, buoyant density, and infectivity of HCV particles (15), thereby highlighting the involvement of extracellular environment components in the lability vs protection of infectious particles.

Here, we hypothesized that some secreted factors could help HCV viral particles to survive in the liver environment and blood circulation. We therefore sought to study the stability of HCV relative to unrelated viruses that can infect hepatocytes and to determine the mechanisms by which secreted factors could protect the infectivity of HCV particles.

## RESULTS

### High instability of HCV intracellular particles at body temperature

**Fig 1:**
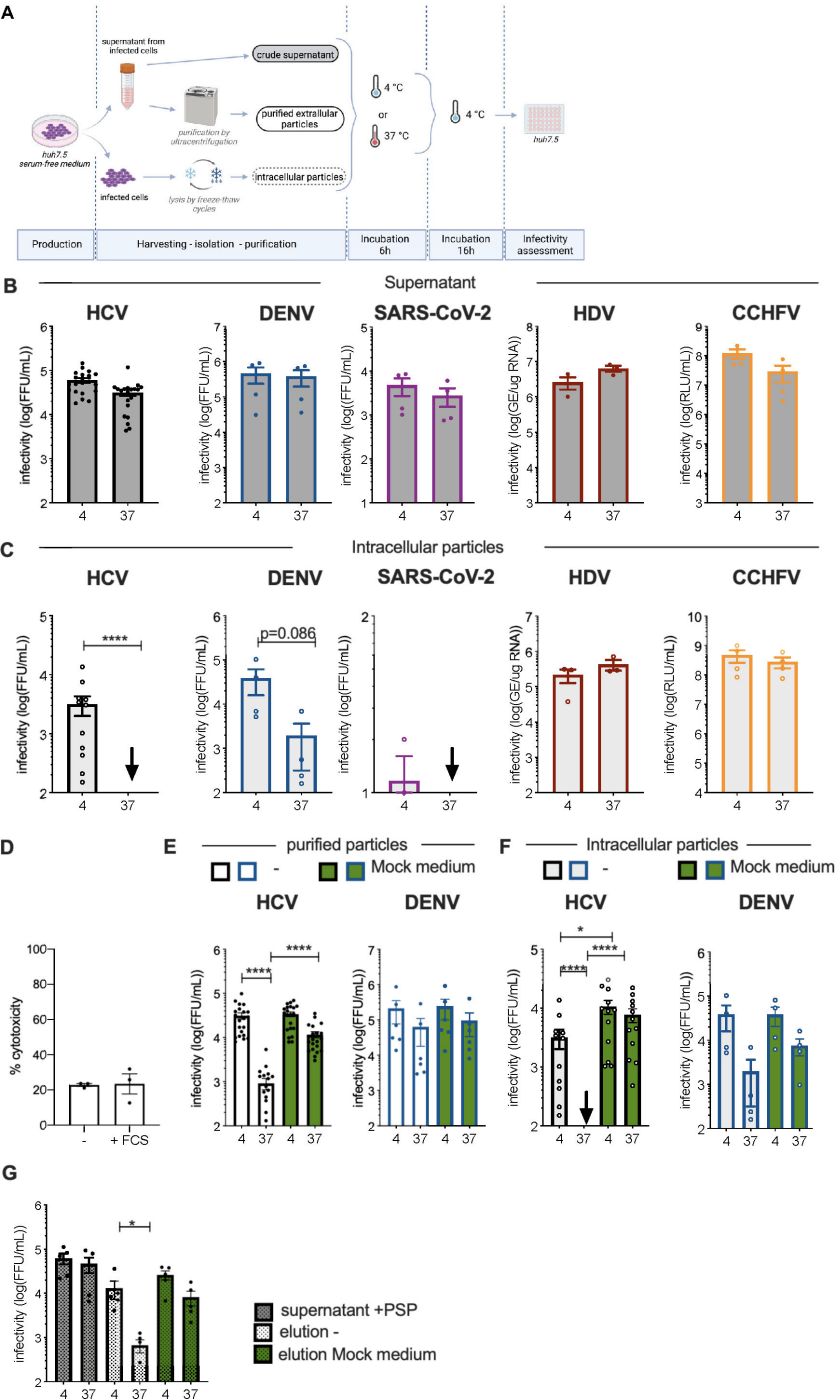
Purified HCV infectious particles are protected from temperature-sensitive degradation by secreted factors. (**A**) Schematic representation of the protocol used to assess stability of infectious particles. Crude supernatants from producer cells were left untreated or were used for purification of viral particles via ultracentrifugation. Cells were lysed by several freeze-thaw cycles to isolate intracellular particles. To assess the stability of virus infectivity, the different preparations of viral particles were either left at 4°C before infection or incubated for 6 h at 37°C (“4” vs “37,” in the figure panels) and then kept at 4°C before infection of naïve Huh-7.5 cells. (**B**) Infectivity of producer cell supernatants treated according to (**A**) containing HCV, DENV, SARS-CoV-2, HDV, and CCFHV particles, as indicated. (**C**) Infectivity of intracellular particles collected from virus-producer cells that were diluted in serum-free medium and treated as in (**A**). (**D**) Cytotoxicity assays of Huh-7.5 cells cultivated in serum-free medium with or without 10% fetal calf serum (FCS), as assessed with CytoTox-Glo kit. (**E**) HCV or DENV extracellular particles were purified by ultracentrifugation. Infectivity of purified extracellular particles diluted in serum-free medium (white bars) or in Mock medium (i.e., a supernatant from Huh-7.5 cells cultivated in serum-free medium for 3 days, green bars) and treated as in (**A**). (**F**) Infectivity of HCV or DENV intracellular particles diluted in serum-free medium (white bars) or in Mock medium (green bars). (**G**) HCV particles harboring a cleavable FLAG tag before E2 sequence, were purified by immunoprecipitation with anti-FLAG antibodies, followed by a cleavage of the FLAG tag by PreScission protease (PSP). Infectivity of crude supernatant cleaved by PSP (gray bars) or purified particles diluted in serum-free medium (white bars) vs Mock medium (green bars) left at 4°C before infection or incubated for 6 h at 37°C and then kept at 4°C before infection. The results are represented as means ± SEM. Each dot in the graphs corresponds to the value of an individual experiment. The arrows represent the infectivity of samples that was below the threshold of detection.

We investigated the stability of infectious particles from different viruses that can replicate in hepatocytes, including HCV, dengue virus (DENV), SARS-CoV-2, hepatitis delta virus (HDV), and Crimean–Congo hemorrhagic fever virus (CCHFV). HCV and DENV particles were produced by electroporation of Huh7.5 cells with *in vitro*-transcribed viral genomic RNAs. SARS-CoV-2 was produced by infection of Huh-7.5 cells with viral stocks. HDV was produced by co-transfection in Huh-7.5 cells with plasmids encoding the HDV RNA genome and the hepatitis B virus (HBV) glycoproteins. For CCHFV, we produced virus-like particles called tecVLPs (22), which have the same structure and composition than full-length CCHFV (23) but whose viral genome segments are replaced by a minigenome segment encoding a reporter gene (nanoLuc). All these viruses were produced in serum-free media to allow their recovery in their most native forms from cell supernatants (Fig. 1A), i.e., by avoiding the influence of factors present in the serum that is typically added to cell culture media. Indeed, for example with HCV, the serum can induce a strong extracellular lipidation of its viral particles via lipid exchanges from lipoproteins and other factors, as shown previously (15).

First, we assessed the infectivity of HCV (Jc1 HCVcc), DENV, SARS-CoV-2, HDV, and CCHFV particles in the crude supernatants of producer cells (i.e., extra-cellular particles) upon a 6-h incubation at 37°C following their harvest (Fig. 1A). Compared to virus supernatants stored at 4°C immediately after harvest, we found that all these viruses were stable in these conditions (Fig. 1B).

Next, we sought to compare the stability of these extracellular particles with their intracellular counterparts. Indeed, since all these viruses are assembled at intracellular membrane [endoplasmic reticulum (ER) for HCV and DENV, ER-Golgi intermediate compartment (ERGIC) for HDV and SARS-CoV-2, and Golgi for CCHFV], we could readily isolate infectious intracellular R)Eviral particles by freeze-thaw lysis cycles for all viruses except for SARS-CoV-2 (Fig. 1C). We then determined the stability of isolated intracellular particles resuspended in serum-free medium upon incubation at 37°C for 6 h. In contrast to HDV and CCHFV intracellular particles that remained stable in these conditions relative to viruses kept at 4°C, we observed a strong loss of infectivity, of >30-fold, for HCV and DENV intracellular particles incubated at 37°C for 6 h (Fig. 1C).

Altogether, our results highlighted a specific feature of *Flaviviridae* intracellular particles that are unstable at 37°C, and gain stability upon secretion in the extracellular medium.

### HCV particles are intrinsically unstable and are protected by secreted factors

Next, we sought to investigate how HCV and DENV become stable upon secretion and egress. As Huh-7.5 cells secrete many factors that may influence virion stability, we sought to evaluate the stability of “purified extracellular viral particles,” i.e., obtained after separation of extracellular viral particles from medium components. Hence, we isolated particles from producer cell supernatants by ultracentrifugation (pellets) and resuspension in serum-free medium (Fig. 1A), and we subsequently analyzed their stability after a 6-h incubation at 37°C. Interestingly, while we observed a moderate decrease of infectivity for DENV of ca. threefold, we detected a profound loss of infectivity for HCV by ca. 34-fold (Fig. 1D, open bars). This suggested that while DENV nascent virions become stable during secretion/egress, the HCV extracellular particles are still highly sensitive to temperature, which could be assessed upon their separation from medium components.

Comparing the stability of HCV particle present in crude supernatants vs in resuspended pellets led us to hypothesize that some secreted cellular factor(s) could stabilize or protect the HCVcc particles. Thus, to investigate the effect of potential stabilizing factors secreted by virus-producer cells on stability of HCV particles, we resuspended purified extracellular or intracellular particles in a “Mock medium,” i.e., consisting of a supernatant of naïve Huh-7.5 cells incubated for 72 h in a serum-free medium, rather than directly in a serum-free medium. No cytotoxicity could be detected during production of Mock media (Fig. 1D), excluding the possibility that the Mock medium could contain some intracellular components just released from dead, lysed cells during production. Strikingly, we found that the Mock medium could fully protect both purified extracellular and intracellular HCV particles from temperature-dependent loss of infectivity (Fig. 1E and F).

To exclude any artifact that could be induced by our procedure of purification of viral particles, we analyzed the stability of HCV particles isolated by immunoprecipitation. Specifically, we used a modified Jc1 virus harboring a FLAG tag on E2 (24) that was further modified by insertion of the human rhinovirus 3C protease cleavage site (25), which, upon cleavage, could avoid interference of the FLAG tag with the infectivity of HCV particles. While the addition of this sequence did not impair infectivity, the combination of antiFLAG IP and cleavage with the PreScission protease (PSP) allowed an efficient and specific recovery of viral particles (Fig. S1). Importantly, using the eluted HCV particles, we confirmed the temperature sensitivity of isolated HCV particles and their protection by Mock medium (Fig. 1G).

Altogether, these results showed that HCV particles are characterized by their high instability at 37°C, which can be overcome by secreted factors.

### HCV-specific infectivity is altered in a genome length- and temperature-dependent manner

**Fig 2:**
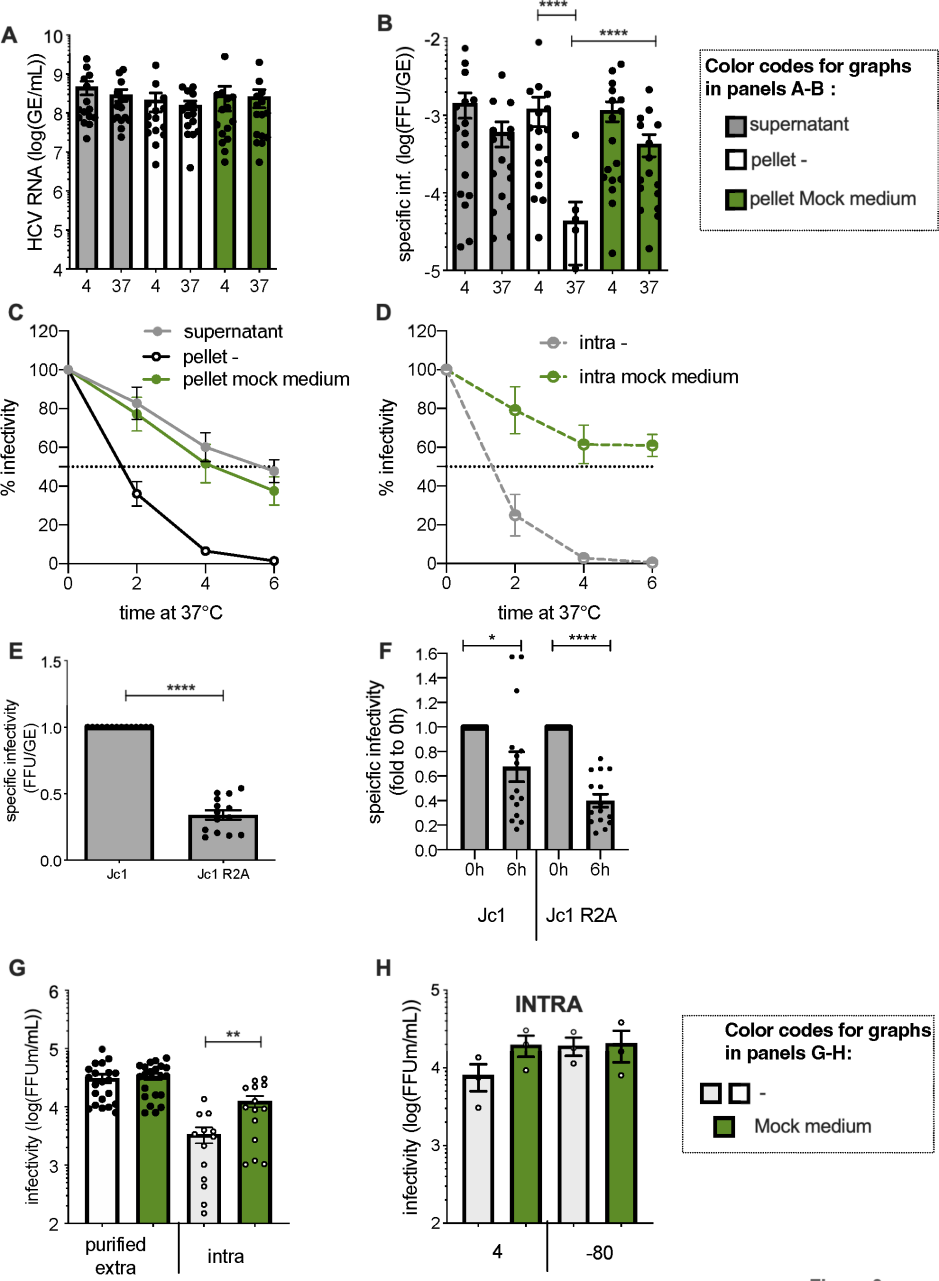
The specific infectivity of purified HCV particles is quickly lost at 37°C. (**A**) Quantification of HCV extracellular RNA isolated from crude supernatants (gray bars) vs from purified extracellular particles diluted in serum-free medium (white bars) or in Mock medium (green bars) and treated as in Fig. 1A. (**B**) Specific infectivity of HCV particles treated as indicated in (**A**). (**C**) Infectivity of extracellular HCV particles from supernatants (gray lines) vs of purified extracellular HCV particles diluted in serum-free medium (pellet –, black lines) or in Mock medium (pellet mock medium, green lines) after incubation at 37°C for the indicated times. (**D**) Same as (**C**) for intracellular particles. (**E**) Specific infectivity of Jc1 vs Jc1 R2A viral particles from crude supernatants of producer cells. (**F**) Specific infectivity of Jc1 vs Jc1 R2A from crude supernatants treated as in Fig. 1A. (**G**) Comparison of infectivity of purified extracellular (plain bars) vs intracellular (dashed bars) particles that were resuspended in serum-free medium (–, white bars) or in Mock medium (green bars) and maintained at 4°C before infection. (**H**) Infectivity of intracellular particles diluted in serum-free medium (–, white bars) or in Mock medium (green bars) and maintained at 4°C vs at −80°C before infection. The results are represented as means ± SEM. For panels (**A and B**) and (**E–H**), each dot in the graphs corresponds to the value of an individual experiment.

To further characterize the decay of infectivity of HCV particles, we titrated the extracellular HCVcc RNAs after 6 h incubation at 37°C. We found that the RNAs isolated from the viral particles from both the cell supernatants and the pellets remained stable (Fig. 2A). This highlighted a loss of HCV-specific infectivity, of ca. 30-fold, rather than a mere degradation of physical viral particles (Fig. 2B). Accordingly, resuspension of pelleted particles in Mock medium prevented the loss of specific infectivity (compare white and green bars in Fig. 2B). Aiming at determining the half-life of HCV infectivity, we harvested particles at different time points after incubation at 37°C. In contrast to the 6 h half-life of secreted HCV particles determined in producer cells supernatants, we found that both purified extracellular and intracellular HCVcc particles exhibited a half-life of ca. 1.5 h (Fig. 2C and D), highlighting an intrinsically high instability of HCV particles at 37°C.

Then, since the viral RNAs remained stable under conditions where infectivity was quickly lost, we wondered if assembling viral particles with a slightly longer genome, which may induce some mechanical constraints at the level of virion conformation, maturation by lipidation, and/or cell entry, could impact HCV stability. Accordingly, we compared virus stability and specific infectivity of the Jc1 HCVcc vs the Jc1-derived R2A virus (26), which expresses a Renilla Luciferase marker that increases HCV genome length by ca. 11%. When determined in producer cell supernatants, we found that the Jc1 R2A virus exhibited a reduced specific infectivity by three- to fourfold (Fig. 2E), indicating that increasing genome length could alter HCV particle infectivity. Moreover, upon incubation for 6 h at 37°C of these viral supernatants, we found that the specific infectivity of the Jc1 R2A HCVcc was further decreased as compared to wt Jc1 virus (Fig. 2F), hence reinforcing the notion that the latter viral particles display high instability.

These results indicated that optimal genome length is a key determinant of HCV stability, which can be assessed at both 37°C and 4°C.

Finally, we sought to investigate the stability of purified extracellular and intracellular particles of HCV at low temperatures, which prevents maturation of viral particles by extracellular lipidation (15). Interestingly, we observed that the Mock medium increased the infectivity of HCVcc intracellular particles maintained at 4°C by over fourfold relative to serum-free medium, in contrast to the purified extracellular particles (Fig. 2G). This suggested that intracellular particles are not fully stable at 4°C and that a destabilizing factor can alter these particles at low temperatures. To confirm this hypothesis, we froze the intracellular particles immediately after their harvest rather than storing them at 4°C before infection. We showed that the HCVcc particles maintained at −80°C were ca. threefold more infectious than those maintained at 4°C, with an infectivity level comparable to particles incubated at 4°C with Mock medium (Fig. 2H).

These results suggested that intracellular particles are unstable at 4°C, which is not the case of extracellular particles, hence suggesting a protecting maturation event during secretion/egress.

### HCV particles are protected by different hepatocyte-specific secreted proteins

**Fig 3:**
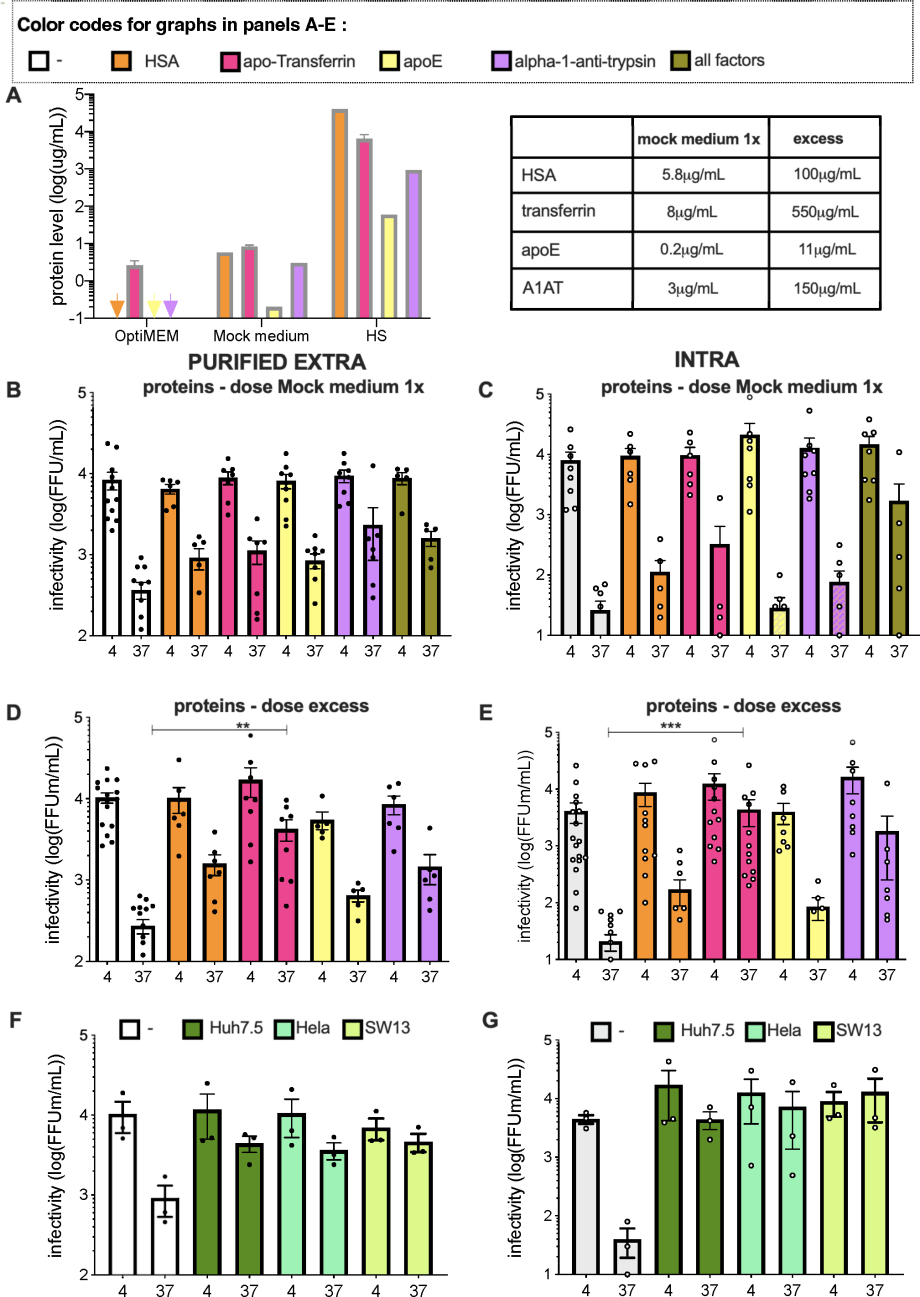
Secreted hepatic factors can protect HCV particles. (**A**) Titers of human serum albumin (orange bars), apo-transferrin (pink bars), apoE (yellow bars), or alpha-1-antitrypsin (violet bars) as determined in serum-free medium (OptiMEM), Mock medium or human serum (HS). The arrows represent the concentration of samples that was below the threshold of detection. (**B**) Infectivity of purified extracellular particles diluted in serum-free medium (white bars) that were or not supplemented with each indicated protein alone at the levels corresponding to Mock medium (**A**) vs with the combination of the four proteins and treated as in Fig. 1A. (**C**) Same as (**B**) for intracellular particles. (**D**) Infectivity of purified extracellular particles diluted in serum-free medium supplemented with each protein alone at a 20-fold higher concentration than in Mock medium. (**E**) Same as (**D**) for intracellular particles. (**F**) Infectivity of purified extracellular particles diluted in serum-free medium (white bars) or in Mock media from Huh7.5, Hela, or SW13 cells, as indicated. (**G**) Same as (**F**) for intracellular particles. For panels (**B–G**), the results are represented as means ± SEM. Each dot in the graphs corresponds to the value of an individual experiment.

The above results prompted us to determine factor(s) expressed by Huh-7.5 cells that may protect HCV particles during their traffic through the secretory pathway. We tested human serum albumin (HSA) and iron-free transferrin (apo-Tf), apolipoprotein E (apoE), and alpha-1-antitryspin (A1AT), which—except for apoE—are proteins that typically produced and secreted by Huh-7.5 cells (Fig. 3A). These proteins were selected because of their high secretion levels (27) and because of their properties, such as iron chelation for apo-Tf and association with HCV particles for apoE (14, 16, 28, 29). Of note, a low level of transferrin was already present in serum-free medium though at a concentration at least fourfold lower than in the Mock medium (Fig. 3A). When tested individually, we found that either factor induced some levels of protection of purified extracellular HCVcc particles, with two- to six-fold increases of viral infectivity after a 6-h incubation at 37°C (Fig. 3B). Furthermore, with the exception of apoE, either factor alone could also induce a significant protection of intracellular particles (Fig. 3C). Interestingly, when we combined these factors together, we found that this could readily protect intracellular particles from instability (Fig. 3B and C). Yet, the protection levels were lower than that provided by the Mock medium (Fig. 1E and F), suggesting that alternative factors might also contribute to stabilize HCV infectivity. Furthermore, we found that Mock media produced from Hela and SW13 cells could also protect purified HCV particles as well as intracellular particles (Fig. 3F and G), suggesting that different or, alternatively, ubiquitous cell-secreted factors can stabilize HCV particles.

Since the above-mentioned factors were individually tested at their concentrations detected in the Mock medium produced by Huh-7.5 cells, which are lower than those detected in human serum (Fig. 3A), we next sought to test them at higher concentrations. Interestingly, HSA, A1AT, and more particularly apo-Tf could efficiently protect purified extracellular HCVcc particles, with 4- to 15-fold increases of viral infectivity after a 6-h incubation at 37°C (Fig. 3D) while apoE remained poorly efficient. For intracellular particles, while HSA and aopE could weakly protect their infectivity, with four- to ninefold increases of viral infectivity after a 6-h incubation at 37°C, we found that A1AT and more particularly apo-Tf could fully prevent the loss of infectivity (Fig. 3E). Interestingly, we found that these factors could also enhance infectivity of HCV intracellular particles maintained at 4°C, by up to fourfold (Fig. 3E).

Altogether, these results suggested that a combination of secreted cellular factors could protect both intracellular and extracellular HCV particles from their intrinsic instability.

### Nonprotected HCV particles are sensitive to oxidation

**Fig 4:**
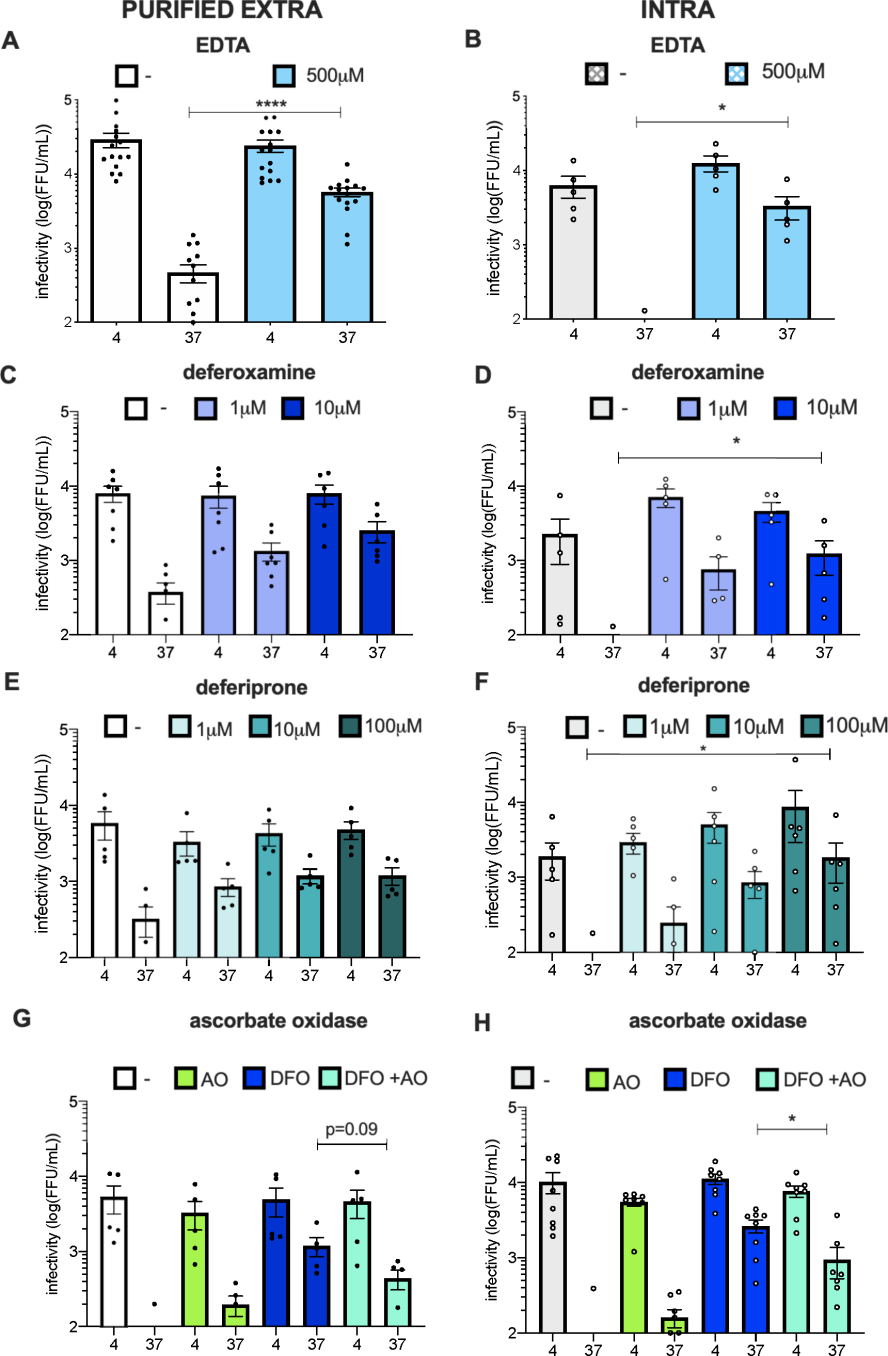
HCV particles are sensitive to iron-induced oxidation. (**A**) Infectivity of purified extracellular particles diluted in serum-free medium (−) vs in serum-free medium supplemented with EDTA (0.5 mM) and treated as in Fig. 1A. (**B**) Same as (**A**) for intracellular particles. (**C**) Infectivity of purified extracellular particles diluted in serum-free medium (−) vs in serum-free medium supplemented with different concentrations of deferoxamine and treated as in Fig. 1A. (**D**) Same as (**C**) for intracellular particles. (**E**) Infectivity of purified extracellular particles diluted in serum-free medium (−) vs in serum-free medium supplemented with different concentrations of deferiprone and treated as in Fig. 1A. (**F**) Same as (**E**) for intracellular particles. (**G**) Infectivity of extracellular particles diluted in serum-free medium (−) vs in serum-free medium supplemented with 10U of ascorbate oxidase (AO) in combination or not with 10 µM of deferoxamine (DFO) and treated as in Fig. 1A. (**H**) Same as (**G**) for intracellular particles. The results are represented as means ± SEM. Each dot in the graphs corresponds to the value of an individual experiment.

As cell culture media contain different ions and since HSA and transferrin can bind several cations including calcium or iron, we hypothesized that the loss of infectivity of HCV particles could be cation-dependent. To test this hypothesis, we supplemented the purified extracellular and intracellular particles with EDTA, which chelates divalent metallic cations. Interestingly, we found that EDTA could protect both purified extracellular and intracellular particles from temperature-dependent degradation (Fig. 4A and B) and that EDTA could also enhance infectivity of intracellular particles kept at 4°C (Fig. 4B). Since transferrin, which can readily protect HCV infectivity (Fig. 3), is specific to iron cation and since our serum-free medium contains Fe^3+^ (ThermoFisher communication), we thought that Fe^3+^ could be involved in inhibition of HCV infectivity. We therefore supplemented purified particles with two iron-specific chelators, i.e., deferoxamine (DFO) and deferiprone (DFP). Interestingly, we observed a dose-dependent protection of both purified extracellular and intracellular particles with either chelator (Fig. 4C through F), suggesting a role of iron cations in the loss of infectivity of infectious particles.

It is well known that iron can induce oxidation under specific environments and more particularly in the presence of ascorbic acid via the Fenton reaction, which results in the formation of ascorbyl radical and ultimately reactive oxygen species (ROS) (30). As our serum-free medium also contains ascorbic acid (ThermoFisher communication), we sought to investigate if infectivity of HCV particles could be specifically affected by oxidation. Hence, we supplemented medium with DFO and ascorbate oxidase (AO), a plant oxidase specific to ascorbate, i.e., the ionic form of ascorbic acid in the medium, to bypass the initial, iron-dependent steps of the Fenton reaction. We observed that AO could overcome the protecting effect of DFO on both purified extracellular and intracellular particles, and could induce a loss of their infectivity (Fig. 4G and H). This indicated that either iron/ascorbate or AO can induce similar alteration of HCV stability.

Finally, as we observed that DENV intracellular particles are unstable at 37°C (Fig. 1C), we sought to address if, like for HCV, EDTA and DFO could stabilize their infectivity. However, in contrast to HCV, such treatment with either EDTA or DFO did not restore the infectivity of intracellular DENV particles (Fig. S2), thus underscoring a different mechanism of instability.

Altogether, these results highlighted that in the absence of cell-secreted protecting factors, HCV particles are sensitive to oxidation.

### Oxidation of HCV particles prevents membrane fusion

We next sought to determine how oxidation could alter HCV infectious particles.

**Fig 5:**
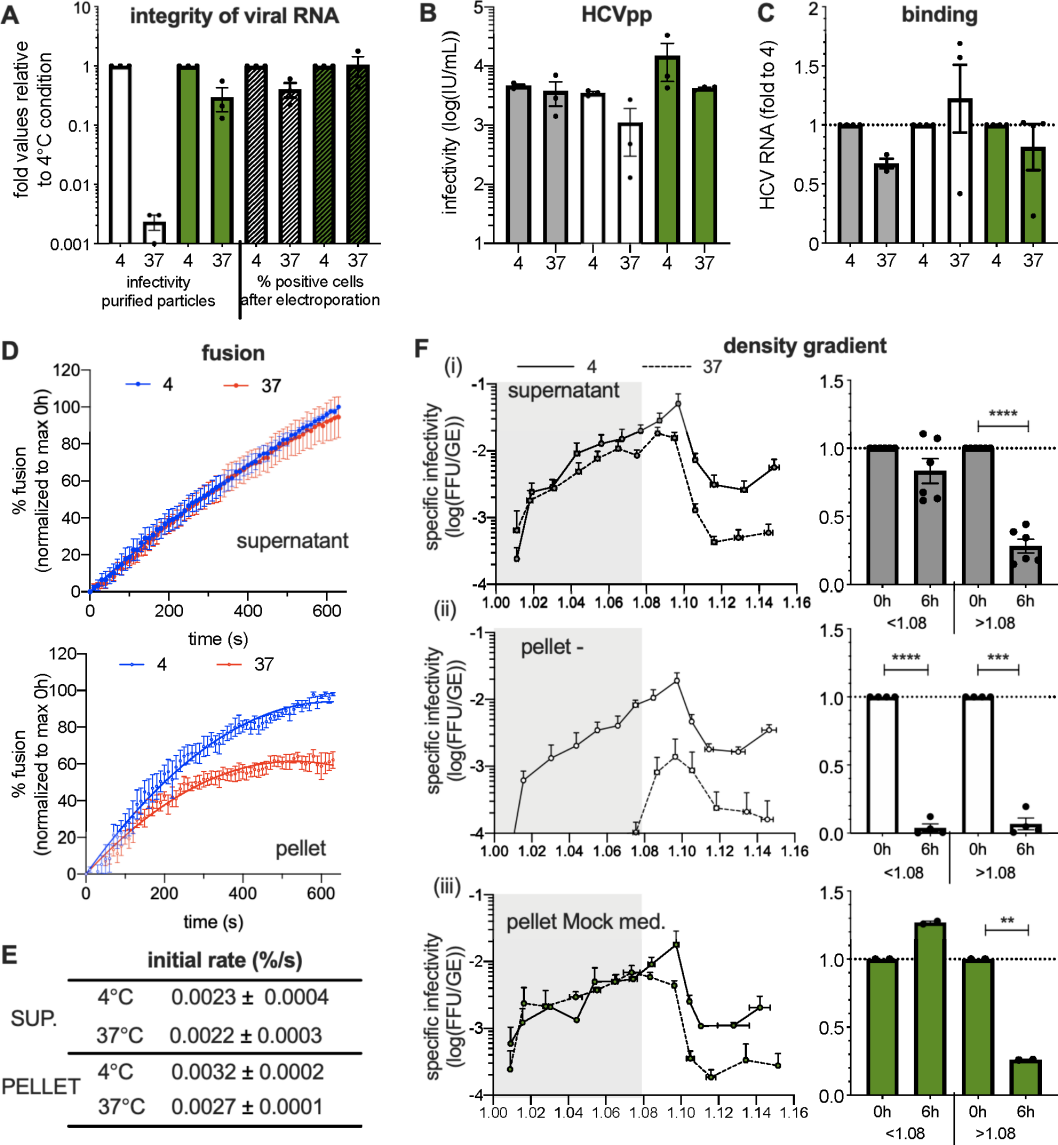
Characterization of HCV properties impaired by oxidation. (**A**) RNA from purified particles diluted in serum-free medium vs in Mock medium and left at 4°C or incubated for 6 h at 37°C were extracted and used for electroporation of naïve cells. The left part of the graph shows the infectivity of the purified particles before extraction of RNA (white bars, viral particles left in serum-free medium; green bars, viral particles incubated in Mock medium) and the right part of the graph shows the percentage of HCV-positive cells after electroporation with extracted RNA (gray bars, percentage of positive cells electroporated with RNA extracted from viral particles left in serum-free medium for 6 h at 4°C or 37°C; green bars, percentage of positive cells electroporated with RNA extracted from viral particles incubated in Mock medium for 6 h at 4°C or 37°C). The data are represented as fold values relative to 4°C incubation. (**B**) Infectivity of HCV pseudoparticles (HCVpp) from crude supernatant of producer cells (gray bars) or purified by ultracentrifugation and diluted in serum-free medium (white bars) or Mock medium (green bars) that were left at 4°C or incubated for 6 h at 37°C. (**C**) Levels of HCV RNA attached to Huh-7.5 cells after 2 h of incubation at 4°C using crude virus producer cell supernatants (gray bars) or purified extracellular particles diluted in serum-free medium (white bars) vs in Mock medium (green bars) that were left at 4°C or incubated for 6 h at 37°C. (**D**) Membrane fusion assays with liposomes using either crude virus producer cell supernatants (top) or purified extracellular particles (bottom) diluted in serum-free medium, left at 4°C (blue lines) or incubated for 6 h at 37°C (red lines). The results show the percentages of fusion relative to Triton X-100-treated liposomes and normalized to the maximum of fusion for 0 h condition for each experiment. (**E**) Values of the initial rates of the fusion curves. (**F**) Specific infectivity of HCV particles detected in fractions from buoyant density gradients of crude virus producer cell supernatant [(i), top graph] or of purified particles diluted in serum-free medium [(ii), middle graph] or in Mock medium [(iii), bottom graph] that were left at 4°C (plain lines) or incubated for 6 h at 37°C (dotted lines). The ratios of specific infectivity of each fraction upon incubation at 37°C vs 4°C were determined (right). The results show the mean of average of these ratios for fractions below and above density of 1.08. The results are represented as means ± SEM. For panels (**A–C**) and F, each dot in the graphs corresponds to the value of an individual experiment.

Our RT-qPCR data suggested that viral RNA is not degraded after incubation of purified viral particles at 37°C (Fig. 2); yet, since qPCR amplifies a small fraction of the genome, we could not firmly conclude that the integrity of the HCV RNA was preserved. Thus, we electroporated naïve Huh-7.5 cells with the same amounts of RNA extracted from purified particles diluted in serum-free medium vs in Mock medium and incubated at 4°C vs 37°C. Indeed, should the genome remain intact, it would allow HCV replication and propagation, which we investigated by flow cytometry to assess expression of the virus core protein. Strikingly, while we observed a strong loss of infectivity of purified HCV particles incubated at 37°C, as expected, we could readily detect HCV-positive cells after electroporation of naïve cells with the RNA extracted from these particles, with an only twofold decrease of HCV-positive cells relative to cells electroporated with RNA extracted from viral particles kept at 4°C (Fig. 5A). These results indicated that the instability of purified HCV particles is not caused by a disruption of their RNA.

Then, to determine if HCV surface glycoproteins were specifically impacted, we tested if HCV pseudoparticles (HCVpp) are sensitive to oxidation. Indeed, HCVpp are retroviral particles harboring functional HCV E1E2 glycoproteins at their surface but no other HCV components (31). Interestingly, we observed that in contrast to HCVcc particles (Fig. 1E), purified HCVpp particles were stable upon incubation at 37°C (Fig. 5B). This suggested that E1 and E2 glycoproteins may not be primarily impacted by the temperature-induced oxidation.

To complement this notion, we measured the attachment to Huh-7.5 cells of purified HCVcc particles after incubation at 37°C. As shown in Fig. 5C, we found that relative to incubation at 4°C, a 6-h incubation at 37°C did not affect the attachment of purified HCVcc extracellular particles to the surface of Huh-7.5 cells.

These results together indicate that E1 and E2 glycoproteins are not impacted by oxidation and, therefore, the loss of infectivity occurs at a post-binding step.

Hence, we sought to determine how oxidation could alter the membrane fusion properties of HCV particles using an *in vitro* assay based on liposomes containing a self-quenched R18 dye (32, 33). We compared the fusion activity of purified extracellular particles relative to those present in crude producer cell supernatants. Strikingly, while incubation at 37°C did not alter the fusion efficiency of HCVcc particles from crude supernatants (Fig. 5D, top), it altered the fusion activity of purified extracellular particles, as shown by the reduction of both the maximal extent at plateau (Fig. 5D, bottom) and the initial rates (Fig. 5D and E) of membrane fusion.

### Lipids of HCV particles are altered by oxidation

That HCVcc particles seemed to be specifically sensitive to oxidation in comparison to other viruses (Fig. 1) or to HCVpp (Fig. 5B) suggested that their instability could be linked to a specific property of authentic HCV particles. Indeed, HCV particles from patients as well as HCVcc are known to be heterogenous in terms of buoyant density (12, 13), which reflects different levels of lipidation of these particles compared to those of HCVpp (34) or DENV (35) particles. We therefore hypothesized that the loss of infectivity due to oxidation could occur for a specific subpopulation of HCV particles. To test this hypothesis, we layered on a density gradient crude HCVcc producer cell supernatants or, alternatively, purified extracellular particles that were incubated beforehand for 6 h at 37°C vs 4°C in serum-free medium. As for crude supernatants, we observed a significant loss of specific infectivity for HCV particles of high densities though they poorly contributed to the overall infectivity (Fig. 5F, upper graphs), whereas there was no substantial loss of infectivity for viral particles of lower densities. Interestingly, for purified extracellular particles, we found a temperature-dependent decrease of specific infectivity of ca. 20-fold for all fractions (Fig. 5F, middle graphs). These results indicated that HCV particles of low densities, which correspond to lipidated virions (15), become specifically unstable upon purification and incubation at 37°C. Moreover, these results suggested that some secreted factors may specifically protect low-density particles. Accordingly, we found that incubation of purified extracellular particles for 6 h in Mock medium resulted in an explicit restoration of the specific infectivity of lipidated particles (Fig. 5F, bottom graphs). Altogether these results indicated that low-density HCV particles are specifically sensitive to oxidation.

**Fig 6:**
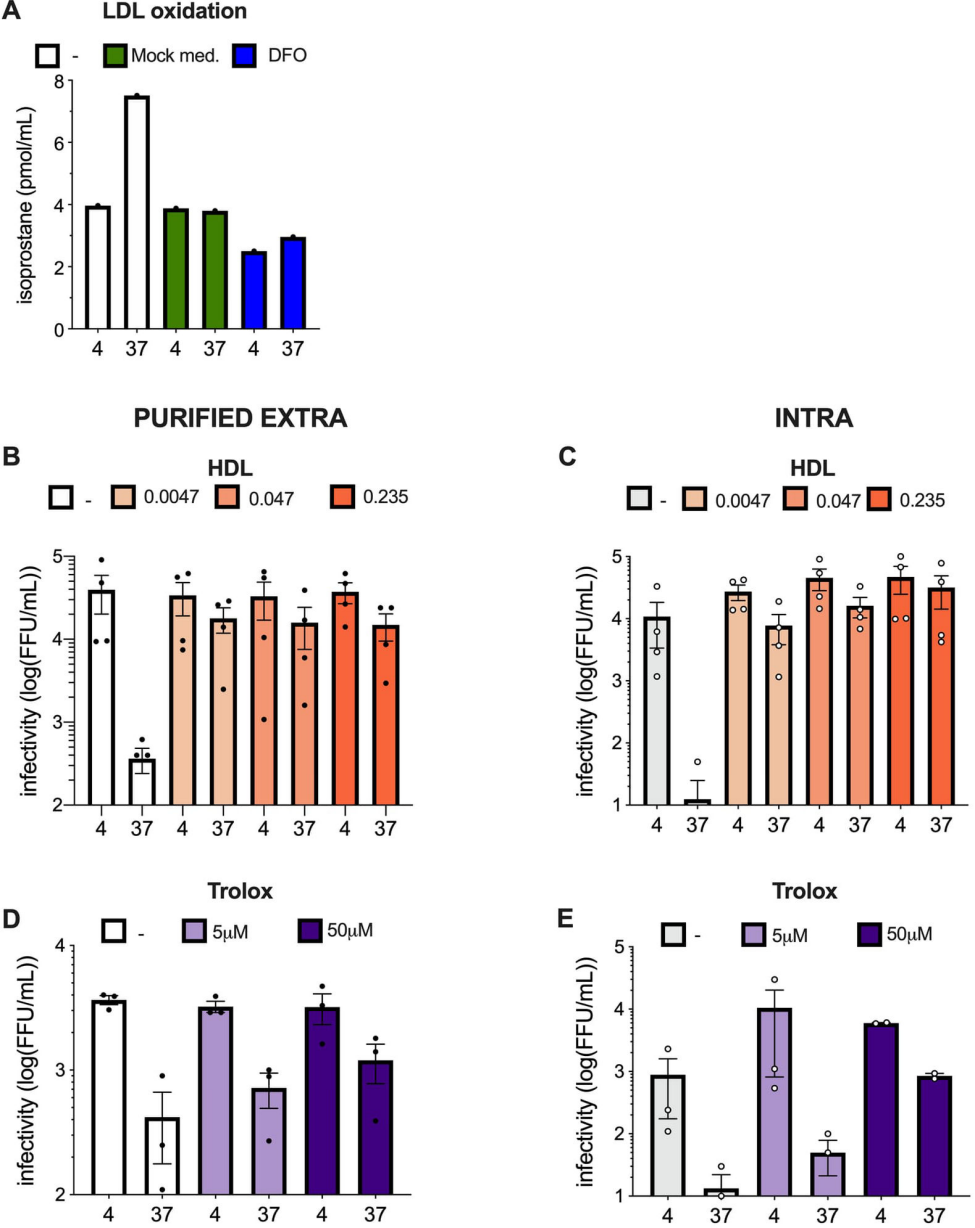
HCV particles are sensitive to lipid peroxidation. (**A**) Amount of isoprostanes found in LDLs diluted in serum-free medium (white bars), Mock medium (green bars), or serum-free medium supplemented with deferoxamine (DFO) (blue bars) and left at 4°C or incubated for 6 h at 37°C. (**B**) Infectivity of purified extracellular particles diluted in serum-free medium (–, white bars) vs in serum-free medium supplemented with different concentrations of HDLs (in g/L) and treated as in Fig. 1A. (**C**) Same as (**B**) for intracellular particles. (**D**) Infectivity of purified extracellular particles diluted in serum-free medium vs in serum-free medium supplemented with different concentrations of Trolox and treated as in Fig. 1A. (**E**) Same as (**D**) for intracellular particles. The results are represented as means ± SEM. Each dot in the graphs corresponds to the value of an individual experiment.

Next, since low-density particles have a high lipid/protein ratio (15), we surmised that lipids (either phospholipids or neutral lipids) of viral particles were specifically sensitive to oxidation, which agrees with the alteration of HCV fusion activity (Fig. 5D and E). We attempted to detect lipid peroxidation by measuring the amounts of isoprostanes, as a marker of lipid oxidation (36). Unfortunately, we could not detect any increase of this marker, even when we induced oxidation with CuSO4, likely owing to too low amounts of lipids in our HCV samples. Thus, to indirectly address if our experimental conditions could induce lipid peroxidation, we used low-density lipoproteins (LDLs) diluted in serum-free medium as a surrogate model of lipidated HCV particles (12), which allowed detection of isoprostanes (Fig. 6A). Interestingly, we detected increased isoprostane levels when these LDL preparations were incubated at 37°C for 6 h (Fig. 6A, white bars). Importantly, we did not detect increase of isoprostane levels when the LDLs were diluted in either Mock medium (Fig. 6A, green bars) or in serum-free medium supplemented with DFO (Fig. 6A, blue bars). Altogether, these results confirmed that our experimental conditions could reflect lipid peroxidation.

Then, to confirm that lipids from viral particles are altered by oxidation, we sought to test if high-density lipoproteins (HDLs), which are known to protect LDLs from lipid peroxidation (37), could protect HCV particles. Hence, we supplemented purified extracellular and intracellular HCV particles with HDLs at different concentrations corresponding to 0.2%, 2%, and 10% of human serum. Interestingly, all HDL doses could readily protect HCV particles from temperature-dependent degradation (Fig. 6B and C). Furthermore, HDL could also protect intracellular particles at 4°C as we also observed an increase of infectivity when HDLs were present at 4°C (Fig. 6C). To complement this approach, we tested the lipophilic antioxidant Trolox, which is a water-soluble analog of vitamin E (38). We found that Trolox could prevent the loss of infectivity of both purified extracellular and intracellular HCV particles upon incubation at 37°C (Fig. 6D and E).

Altogether, these results confirmed that lipids of HCV particles are sensitive to peroxidation.

Finally, since HCVcc particles with a longer genome (Jc1 R2A HCVcc) appeared to be more unstable than wt virus (Fig. 2E and F), we surmised that this difference could be linked to a differential reactivity to oxidation of their lipidated particles; the characterization of which may provide hints to understand mechanisms evolved by HCV to mitigate the impact of oxidation after viral production.

**Fig 7:**
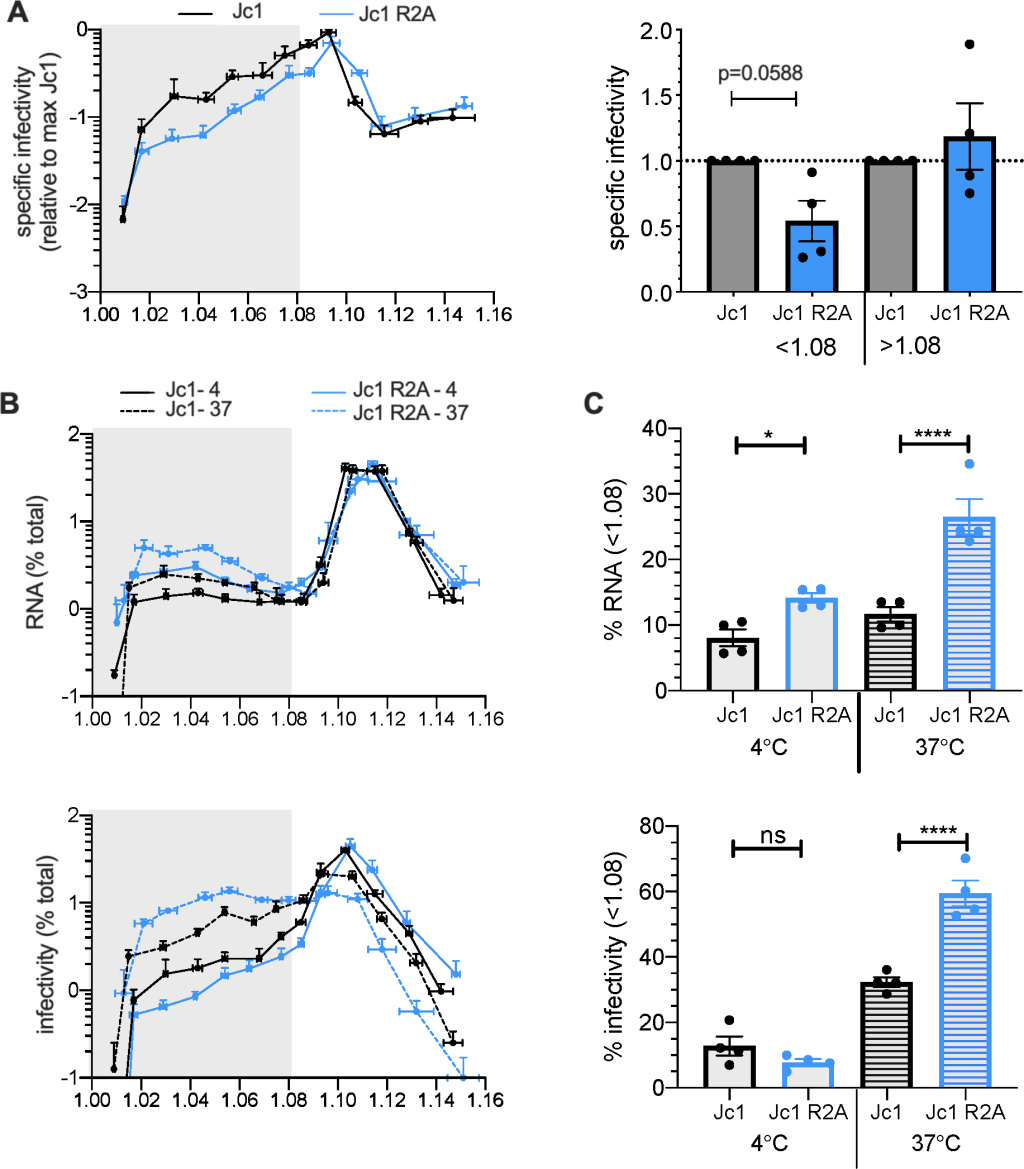
The size of the HCV genome influences densities and stability of viral particles. (**A**) Specific infectivity of Jc1 (black line) vs Jc1 R2A (blue line) detected in fractions from buoyant density gradients of crude supernatant. The ratios of specific infectivity of each fraction upon incubation at 37°C vs 4°C were determined (right). The results show the mean of average of these ratios for fractions below and above density of 1.08. (**B**) Viral RNA (top) or infectivity (bottom) of Jc1 (black lines) vs Jc1 R2A (blue lines) particles detected in fractions from density gradients of crude virus producer cell supernatant after incubation at 4°C (plain lines) and 37°C (dotted lines). (**C**) The sum of percentage of RNA (top graph) or infectivity (bottom graph) in fractions below 1.08 from (**B**) is represented. The results are represented as means ± SEM. Each dot in the graphs corresponds to the value of an individual experiment.

First, we compared the stability of Jc1 R2A HCVcc with parental Jc1 virus through their analysis in density gradients. We found that compared to Jc1 HCVcc, the Jc1 R2A virus exhibited a twofold reduction of specific infectivity in its low-density fractions (Fig. 7A), indicating that Jc1 R2A low-density particles are more unstable than those of Jc1. Interestingly, compared to the Jc1 HCVcc, we also found that proportionally, the Jc1 R2A virus displayed more abundant RNA levels—reflecting viral particles—in low-density fractions (Fig. 7B, plain lines in upper graphs), which, unexpectedly, were associated to a loss of infectivity (Fig. 7B, plain lines in lower graphs). That the Jc1 R2A virus, which seems more efficiently lipidated than Jc1 HCVcc particles, had specifically lost infectivity of its lipidated fractions agreed with the hypothesis that lipidated particles are more sensitive to oxidation.

Thus, to further explore the basis of the difference between either virus for lipidation vs oxidation of viral particles, we allowed them to become lipidated by incubating their supernatants for 6 h at 37°C, which induces partial virion lipidation by lipids released from Huh-7.5 cells (Fig. S3). Accordingly, we detected significantly higher levels of viral particles in low-density fractions for both Jc1 and Jc1 R2A viruses (Fig. 7B, upper graphs). Yet, while this increase of RNA levels in lipidated fractions was similar between Jc1 and Jc1 R2A, we observed a greater increase of infectious titers for the Jc1 R2A virus (Fig. 7B, lower graphs), suggesting a higher dynamic of lipidation for this latter virus.

Altogether, these results indicated that lipidation as well as rates of lipid exchanges are linked to the stability of HCV and that genome length may modulate the above.

Overall, these results suggested that HCV particles lipids are specifically sensitive to oxidation, leading to an altered fusion capacity and thus a loss of entry capacities and infectivity.

## DISCUSSION

Summarizing, here we show first that HCV particles are intrinsically labile, which drastically alters their specific infectivity by oxidation of their lipids, and second that they are protected from lipid oxidation by secreted cellular proteins, which can preserve their membrane fusion capacity and overall infectivity.

Interestingly, our results show that HCV particles are sensitive to oxidation-mediated degradation in contrast to other tested viruses that infect hepatocytes, including a closely related member of the *Flaviviridae* family, i.e., DENV. We show that this property is due to the unique feature of HCV particles, i.e., their association with neutral lipids such as triglycerides and cholesterol esters, which results in their peculiar low density relative to other viruses (11 – 16). In this respect, HCV particles have a lipid composition closer to LDL than to other enveloped viral particles such as, e.g., HIV particles (24), which induces their specific and high sensitivity to oxidation, as shown in our report. Of note, we also show that HCVpp are not sensitive to oxidation. While both HCVpp and HCVcc particles harbor E1 and E2 glycoproteins at their surface, the lipid composition of either viral particle is dissimilar (34), which is due to their different assembly sites [ER for HCVcc and multi-vesicular bodies (MVBs) for HCVpp (39)] and to specific acquisition of neutral lipids by HCVcc (15, 40). This explains the link between the unique lipidic composition of authentic HCV particles and their sensitivity to oxidation.

Our results reveal that oxidation alters the fusogenicity of purified HCV particles. This agrees with a previous report showing that knock-down of GPx4, a key protector of intracellular lipid peroxidation, resulted in secretion of HCV particles with altered fusogenicity (41). While in this report, lipid peroxidation was induced inside the cells, which altered viral particles during their assembly and/or secretion, here we show that lipid oxidation could also impair fusogenicity of particles after their secretion and we highlight both the targeted viral components and the mechanisms of protection developed by HCV particles. Typically, membrane fusion involves activation of membrane surface proteins, such as viral glycoproteins, and mixing of lipids from outer and inner membrane layers (42). Since our data indicated that HCVpp are not sensitive to oxidation and that cellular binding of HCVcc was not impaired (Fig. 5), this led us to propose that fusion impairment was caused by a defect of the lipid bilayer rather than to an alteration of the viral surface glycoproteins themselves. Formation of ROS that are free radicals that can damage organic macromolecules may occur as a product of the Fenton reaction (30), which can be initiated by the presence of ascorbate and iron in the environment. Then, ROS can react with cholesterol or with unsaturated fatty acids via their carbon–carbon double bonds and create hydroperoxide groups. This could alter properties of the viral membrane such as its fluidity or curvature, which may alter its membrane fusion properties (43). Interestingly, light-activated membrane-targeting singlet oxygen (^1^O2) generators, leading to ROS formation, were developed as broad-spectrum antiviral compounds (43). One of such compounds, LJ001, was shown to inhibit the membrane fusion properties of HIV and HCV particles by altering lipid packing and membrane fluidity, confirming the link between lipid oxidation and viral fusion (44 – 46). More precisely, at the molecular level, we could speculate on the one hand, that the oxidized lipids would cluster into microdomains in order to protect the resulting polar groups from hydrophobic repulsion and on the other hand, that the surface of viral particles would inflate owing to the addition of hydroperoxide groups, both events leading to an alteration of the fusogenicity of the membrane.

Importantly, we found that several serum proteins could protect purified HCV particles from oxidation, namely apo-Tf, HSA, and A1AT that are typically secreted by hepatocytes and by lipid-free apoE that is present in serum, albeit at variable efficiency (Fig. 3), although it appears that the mechanism(s) may also involve alternative factors. As these proteins display various and non-related functions, we can only speculate about the different mechanisms by which they could induce protection of viral particles. First, transferrin could protect HCVcc particles by chelating iron cations, which is its main function, and which would therefore prevent the Fenton reaction and subsequent oxidation to occur. Second, HSA exhibits antioxidant properties as it can display binding sites for several metal ions, including iron, but also as its Cysteine at position 34 can be oxidized (47), which could therefore prevent reaction of ROS with viral particles. Indeed, HSA is primarily detected in its reduced form although about 30–40% could be variably oxidized with either reversible or irreversible modification (48). Yet, we cannot exclude the possibility that a previously described direct interaction between HSA and HCV particles (15) could, by ‘shielding’ the latter, reduce their oxidation. Third, A1AT was also shown to display antioxidant properties via its methionine residues (49), which could be oxidized instead of viral particles. Finally, apoE also has antioxidant properties (50) probably by scavenging radicals (51). Yet, we can also speculate that it could protect the viral lipid bilayer from oxidation by making a physical barrier, since apoE was shown to bind to HCV particles after secretion (28, 29). Finally, we could not exclude that alternative antioxidant factors secreted by hepatocytes or other cell types may also contribute to protect HCV particles form oxidation.

Our data reveal a different sensitivity to oxidation between intracellular particles and extracellular particles. Indeed, the loss of infectivity was ca. 10-fold higher for the former, as compared to the latter (Fig. 2). Moreover, freezing at −80°C (Fig. 2H) or treatment of the former particles immediately put on ice upon harvest with the above hepatocyte factors (Fig. 1E, 2G, and 3E), chelators (Fig. 4B, D, and F), or antioxidants (Fig. 5I) could increase their infectivity by up to fivefold, as compared to the latter particles. One explanation could be due to a poorly characterized maturation process of HCV particles since HCV intracellular and extracellular particles seem to exhibit different physico-chemical properties, such as pH sensitivity (52) or buoyant density (15, 53), though the latter property may not fully explain the increased sensitivity to oxidation since intracellular particles are not or much less lipidated than extracellular particles (15). On the other hand, intracellular particles are likely to represent more native forms of infectious viral particles, i.e., before they can acquire neutral lipids, which readily occurs after egress (15). Indeed, when extracellular particles acquire neutral lipids, they also incorporate some serum proteins, such as, e.g., apoE while they accumulate in the extracellular medium (28, 29), which results in an equilibrium between factors protecting (e.g., apoE) vs sensitizing (e.g., lipids) virions to oxidation. Note that such events may also partly occur during traffic of viral particles through the secretion pathway, after their biogenesis in the endoplasmic reticulum (11). As virions traffic via organelles that could also be used by secreted hepatocyte factors (54, 55), this co-trafficking is likely to increase their encountering and mutual interrelations, which may ultimately protect viral particles from oxidation.

Thus, altogether, our results highlight a new crossroad between HCV particles and serum components, such as proteins, but also lipoproteins since some secreted factors that promote antioxidative protection of viral particles, as shown in this report, were also shown to condition physico-chemical properties of HCV particles, such as their buoyant density. For example, HSA mediates lipidation of the viral particles by lipoproteins in the serum (15). On the other hand, secreted lipases, such as lipoprotein lipase and hepatic triglyceride lipase, have been shown to increase the density of HCV particles by removing lipids (19). Yet, whether lipase activity may promote HCV oxidation remains to be shown. Besides, other serum components can influence HCV particles infectivity and immune evasion. Indeed, HDLs as well as some lipid-free apolipoproteins, such as ApoC1 and ApoE, can promote HCV entry and inhibit neutralizing antibodies (56 – 58). In contrast, oxidized LDLs could decrease HCV entry (59).

Finally, our data underscore an intriguing relation between stability of HCV particles and the size of the viral genome they harbor (Fig. 6). Indeed, while a 10% increase of the size of the HCV RNA had no impact on viral replication, it strongly altered the infectivity and stability of the particles. We can only speculate at this stage on potential explanations since, clearly, additional experiments that were beyond the scope of this report are needed to fully understand the mechanisms that regulate these events. Interestingly, an allometric relationship between viral genome length and virion volumes was identified (60), suggesting that genome size could directly influence the composition or the architecture of particles and how viruses have evolved to reach the optimal genome size. We can therefore propose that the artificial extension of genome size may result in particles containing more phospholipids and/or cholesterol, as the inside of particles would need to expand. Indeed, any increase of genome size may require more core proteins to protect the HCV RNA, hence resulting in a larger particle volume and therefore requiring more fatty acids to envelope such a nucleocapsid. Yet, although more phospholipids could be recruited on virion, this may not necessarily translate in more glycoproteins being incorporated since the level of glycoproteins at the surface of viral particles is likely to be regulated by other finely tuned processes that involve, e.g., delayed cleavage between E2 and p7 (10), as well as preformed nucleocapsids (61), before envelopment steps. Overall, this could result in larger amounts of fatty acids in and/or on virions that would be more accessible to oxidation, thereby decreasing the stability of HCV particles.

## MATERIALS AND METHODS

### Cells

Huh-7.5 cells (a kind gift from Charles Rice, Rockefeller University, New York, USA), Huh-7-NTCP cells (62), and HEK-293T kidney cells (ATCC CRL-1573) were grown in Dulbecco’s modified minimal essential medium (DMEM, Invitrogen, France) supplemented with 100 U/mL of penicillin, 100 µg/mL of streptomycin, and 10% of FCS (A10111-1098/RFR1103719P, GE Healthcare).

### Plasmids

pFK-JFH1/J6/C-846 and pFK_i389-Rluc_2a_Core_3' plasmids encoding full-length Jc1 HCV, with or without reporter renilla luciferase, respectively, were kind gifts from R. Bartenschlager (Heidelberg University, Germany). pFK-JFH1/J6/C-846_FLAG-E2 was a kind gift from T. Pietschmann (Twincore, Germany) and was used to generate pFK-JFH1/J6/C-846_FAG_3C harboring the human rhinovirus 3C protease cleavage site between FLAG peptide and E2 sequence, by PCR mutagenesis (oligonucleotide sequences are available upon request).

The constructs encoding wild-type CCHFV strain IbAr10200 L polymerase (pCAGGS-V5-L), N nucleoprotein (pCAGGS-NP), M segment (pCAGGS-M), T7 RNA polymerase (pCAGGS-T7), nLuc-expressing minigenome flanked by L NCR under the control of a T7 promotor (pSMART-LCK_L-Luc), and an empty vector (pCAGGS) were described previously (22, 63).

The constructs encoding HDV Ag (pSVLD3) and HBV envelope glycoproteins (pT7HB2.7) were described previously (64). The constructs encoding full-length DENV-2 strain New Guinea C (NGC) pDVWS601 was described previously (65).

### Reagents

Human serum albumin (HSA) fraction V (Sigma-Aldrich), apolipoprotein E from human plasma (Sigma-Aldrich), human apo-transferrin (Sigma-Aldrich), and alpha-1-antitrypsin from human plasma (Sigma-Aldrich) were used at concentration indicated in Fig. 3. Ascorbate Oxidase (AO) from Cucurbita sp. (Sigma-Aldrich), Deferiprone (DFP) (Sigma Aldrich), deferoxamine mesylate (DFO) (Sigma Aldrich), EDTA (Invitrogen), Trolox (Sigma Aldrich), and purified human HDLs (Sigma Aldrich) were used at the indicated concentrations.

### Protein level evaluation

Human apoB and apoE concentrations were measured in OptiMEM and in Huh-7.5 cell supernatant (Mock medium) using ELISA (Mabtech) according to manufacturer’s instructions. HSA concentrations were measured using Cobas C501 analyzer (Roche Applied Science). Human transferrin and alpha-1-antitrypsin concentrations were evaluated by quantitative western blot using a standard curve made with purified apo-transferrin and alpha-1-antitrypsin.

### Cytotoxicity measurement

The cytotoxicity of cells cultured in serum-free medium was assessed 72 h post-seeding using Cytotox-Glo Cytotoxicity Assay (Promega) according to the manufacturer’s protocol.

### Production of HCVcc particles

HCVcc particles were produced as described previously (15). Electroporated Huh-7.5 cells were grown in serum-free medium (OptiMEM, Invitrogen). Intracellular infectivity was determined as described previously from HCVcc producer cells following four freeze/thaw cycles. Virus-containing supernatants were collected at 3 days post-electroporation and clarified through a 0.45-µm filter (Corning Inc, Corning, NY, USA). Viral stocks were titrated on Huh-7.5 cells by immunostaining with anti-NS5A 3E10 (kind gift from C. Rice).

### Production of DENVcc particles

Viral stocks of the DENV-2 strain New Guinea C (NGC) (AF038403) was produced using *in vitro* RNA transcripts prepared from DENV-2 infectious plasmid clone pDVWS601 plasmid (65). Briefly, plasmid was linearized with XbaI (New England Biolabs, Ipswich, MA, USA) and RNA transcripts were produced and purified using mMESSAGE mMACHINE T7 Kit (Ambion/ThermoFisher Scientific, Waltham, MA, USA). RNA transcripts were introduced into Huh-7.5 cells by electroporation (using 5 µg RNA for 6 × 10^6^ cells per electroporation). Virus-containing supernatants were collected at 3 days post-electroporation and clarified through a 0.45-µm filter (Corning Inc, Corning, NY, USA). Viral stocks were titrated on Huh-7.5 cells.

### Production of SARS-CoV-2 particles

SARS-CoV-2 particles were amplified on Vero E6 cells as described previously (66). SARS-CoV-2 virions were then produced in serum-free OptiMEM by infection of Huh-7.5 cells using this viral stock at MOI = 0.1. Supernatants containing SARS-CoV-2 particles were clarified by centrifugation for 10 min at 5,000 × *g* at 72 h post-infection. For titration, Huh-7.5 cells were infected with different dilutions of virus. At 6 h post-infection, the medium was replaced by DMEM/10% FCS containing 1.2% of carboxymethylcellulose (Sigma). Cells were fixed at 72 h post-infection and were immunostained with SARS-CoV-2 nucleocapsid antibodies (Sino Biological).

### Production of HDV particles

HDV virions were produced in serum-free OptiMEM culture media by co-transfection of Huh-7.5 cells with a 1:1 mixture of the pSVLD3 plasmid encoding the HDV genome and the pT7HB2.7 plasmid encoding the HBV glycoproteins using GeneJammer transfection reagent (Agilent), as described previously. Following addition of the transfection mixture, the medium was removed after a 6-h incubation and replaced with fresh serum-free medium. HDV producer cells were maintained under serum-free conditions for 10 days, with the medium replaced every second or third day. Supernatants containing HDV virions were clarified by microfiltration through 0.45 µM filters. Infection assays were performed using Huh-7-NTCP cells. Huh-7-NTCP cells were incubated with HDV overnight and the infection medium was replaced by William’s E medium (WME) (Gibco, France) supplemented with non-essential amino acids, 2 mM L-glutamine, 10 mM 4-(2-hydroxyethyl)-1-piperazineethanesulfonic acid (HEPES) buffer, 100 U/mL of penicillin, 100 µg/mL of streptomycin, 10% fetal bovine serum, and 2% of DMSO. Infected Huh-7-NTCP cells were analyzed at day 9 post-infection by RT-qPCR (62).

### Production of CCHFV tecVLP particles

Huh-7.5 cells were washed two times with OptiMEM and seeded overnight in the same medium in 10cm dish. Cells were transfected with 3.6µg of pCAGGS-V5-L, 1.2µg of pCAGGS-NP, 3µg of pCAGGS-M or pCAGGS, 3µg of pCAGGS-T7, and 1.2µg of pSMART-LCK_L-Luc, using GeneJammer transfection reagent (Agilent), as described previously (67). The transfection medium was replaced 6 h post-transfection. Seventy-two hours post-transfection, supernatant was harvested and filtered through a 0.45-µm filter. For titration, Huh-7.5 cells were pre-transfected using 2.4 µg of pCAGGS-V5-L and 4.8 µg of pCAGGS-NP using GeneJammer transfection reagent. The transfection medium was replaced at 6 h post-transfection and cells were seeded in 24-well plates in OptiMEM. Twenty-four hours post pre-transfection, cells were infected and then lysed after 24 h using Passive Lysis Buffer 1x (Promega), incubated 15 min at room temperature, and the nLuc activity was assessed using the Nano-Glo Luciferase Assay System (Promega) and a Mithras LB 940.

### Production of HCV pseudoparticles

To generate HCVpp, 293T cells were transfected with expression vectors encoding the viral components, i.e., E1E2 glycoproteins, retroviral Gag-Pol proteins, and packaging-competent GFP containing retroviral transfer vectors as described previously (31).

Medium (6 mL/plate) was replaced 16 h after transfection. Supernatants containing the pseudo-particles were harvested 24 h later, filtered through 0.45 µm pore-sized membranes, and used in infection assays. Target Huh-7.5 cells were seeded in 48-well plates at a density of 1.5 × 10^4^ cells per well and incubated overnight at 37°C. Dilutions of viral supernatants containing the pseudo-particles were added to the cells and the plates were incubated for 6 h. The supernatants were removed, and the cells were incubated in regular medium for 72 h at 37°C. The infectious titers, expressed as infectious units (IU) per milliliter, were deduced from the transduction efficiencies, determined as the percentage of GFP-positive cells measured by FACS analysis (BD Canto).

### Purification of viral particles by ultracentrifugation

Cell supernatant containing viral particles were harvested 72 h after electroporation and concentrated 67 times by ultracentrifugation at 25,000 × *g* in a Beckman SW41 rotor for 105 min at 4°C, followed by resuspension in PBS for 20 min at 4°C, before further analysis.

### Purification of viral particles by immunoprecipitation

Supernatant from cells electroporated with RNA encoding for Jc1 FLAG_3C were incubated 6 h with antiFLAG agarose beads (Sigma-Aldrich) at 4°C under continuous agitation. The complexes were washed three times with PBS before overnight incubation with PSP (Sigma-Aldrich). The supernatant containing eluted particles, after cleavage of the FLAG peptide, was then used for stability measurement.

### Stability measurement

Stability was measured by diluting purified particles or intracellular particles 50-fold in serum-free medium supplemented, or not, with different components or with Mock medium. The samples were split in two: one part was incubated for 6 h at 37°C in the CO_2_incubator and the other part was left at 4°C. The samples were then stored at 4°C for 16 h before measuring infectivity or RNA or before using the particles for indicated assays.

### Determination of viral RNA by RT-qPCR

HCV RNAs were extracted (TRI Reagent, Euromedex), reverse transcribed (iScript cDNA synthesis kit, Bio-Rad), and quantified (FastStart Universal SYBR Green Master kit, Roche Applied Science) on an Applied StepOne Real-Time PCR apparatus. HCV-specific primers (U147: 5′-TCT GCG GAA CCG GTG AGT A and L277: 5′-TCA GGC AGT ACC ACA AGG C). As an internal control of extraction, an exogenous RNA from the linearized Triplescript plasmid pTRI-Xef (Invitrogen) was added into the supernatant prior to extraction and quantified with specific primers (Xef-1a 970L20: 5′-CGA CGT TGT CAC CGG GCA CG and Xef-1a 864U24: 5′-ACC AGG CAT GGT GGT TAC CTT TGC). For binding assay, GAPDH level was quantified using specific primers (GAPDH 83U: 5′-TGGAAGATGGTGATGGGATTTC and GAPDH 287L: 5′-AGGTGAAGGTCGAGTCAACG).

### Binding of HCVcc particles to cells

Huh-7.5 cells were plated in 12 well previously coated with collagen. One day later, cells were incubated with 1 mL of viral supernatant or purified particles for 2 h and washed twice with PBS supplemented with calcium and magnesium (0.7 mM CaCl2, 0.25 mM MgSO4). Total RNA cell lysates were extracted to quantify the level of HCV and GAPDH gene by RT-qPCR.

### Iodixanol density gradient of HCVcc particles

One milliliter of samples (supernatant or diluted pellet) was layered on top of a 0–30% continuous iodixanol gradient (Optiprep; Axis-Shield). Gradients were centrifuged for 16 h at 32,000 rpm in a SW41 swinging rotor at 4°C using an Optima L-90 K Beckmann centrifuge. Fifteen fractions of 750 µL were collected from the top and were analyzed for virus infectivity and viral RNA copies.

### Fusion of HCVcc particles with liposomes

HCVcc/liposome lipid mixing assays were performed as previously described for HCVpp and HCVcc (32, 33). All liposomes were large unilamellar vesicles (100 nm) consisting of phosphatidylcholine (from egg yolk; Sigma Aldrich), cholesterol (Sigma Aldrich), and Octadecyl rhodamine B chloride (R18; Invitrogen) (65:30:5 mol%). R18-labeled liposomes were obtained by mixing R18 and lipids as ethanol and chloroform solutions. Lipid mixing was assessed essentially as described and monitored as the dequenching of R18. Briefly, HCVcc particles treated as indicated were added to a cuvette containing R18-labeled liposomes (final lipid concentration, 15 µM). After temperature equilibration at pH 7.4 for 2 min, fusion was initiated by adding an appropriate volume of diluted HCl to the cuvette, and kinetics were recorded using a dual-channel PicoFluor hand-held fluorimeter (Turner Biosystems, Sunnyvale, CA, USA), operated under the “rhodamine” channel (excitation and emission wavelengths 540 ± 20 and >570 nm, respectively). Maximal R18 dequenching was measured after the addition of 0.1% Triton X-100 (final concentration) to the cuvette. Initial rates of fusion were taken as the value of the slope of the tangent, drawn to the steepest part of the fusion kinetics. Final extent of lipid mixing was the value obtained when fluorescence reached a plateau.

### Electroporation of purified RNA from particles

Particles purified by ultracentrifugation were used for standard stability measurement. Samples were then concentrated using Vivaspin columns with a cut-off of 100 kDa. Viral RNA from these particles was then extracted and the amounts of copies were determined by RT-qPCR according to our protocol (see above). Same amount of viral RNA was then electroporated in Huh7.5 according to the classical protocol. Seventy-two hours post-electroporation, cells were harvested and the percentage of HCV-positive cells was determined by flow cytometry using an anti-NS5A antibody as described previously (10).

### Statistical analysis

Significance values were calculated by applying Mann–Whitney or Kruskal–Wallis and Dunn’s multiple comparison tests using the GraphPad Prism nine software (GraphPad Software, USA). *P* values under 0.05 were considered statistically significant and the following denotations were used: ****, *P* < 0.0001; ***, *P* < 0.001; **, *P* < 0.01; *, *P* < 0.05; ns (not significant), *P* > 0.05.

## Data Availability

The data sets generated during the current study are available from the corresponding author upon reasonable request.

## SUPPLEMENTAL MATERIAL

Fig. S1 mbio.01549-23-s0001.tif Addition of a cleavage site between FLAG and E2 sequences.

Fig. S2 mbio.01549-23-s0002.tif No effect of DFO and EDTA on intracellular DENV particles.

Fig. S3 mbio.01549-23-s0003.tif Effect of knock-down of apoB on density gradient of HCVcc.

Supplemental Legends mbio.01549-23-s0004.docx Supplemental figure legends.

ASM does not own the copyrights to Supplemental Material that may be linked to, or accessed through, an article. The authors have granted ASM a non-exclusive, world-wide license to publish the Supplemental Material files. Please contact the corresponding author directly for reuse.
