# Supplementary figures and images for: Low-density hepatitis C virus infectious particles are protected from oxidation by secreted cellular proteins
Source: mBio. 2023 Sep 6;14(5):e01549-23. doi: 10.1128/mbio.01549-23 (PMC10653866; doi:10.1128/mbio.01549-23)

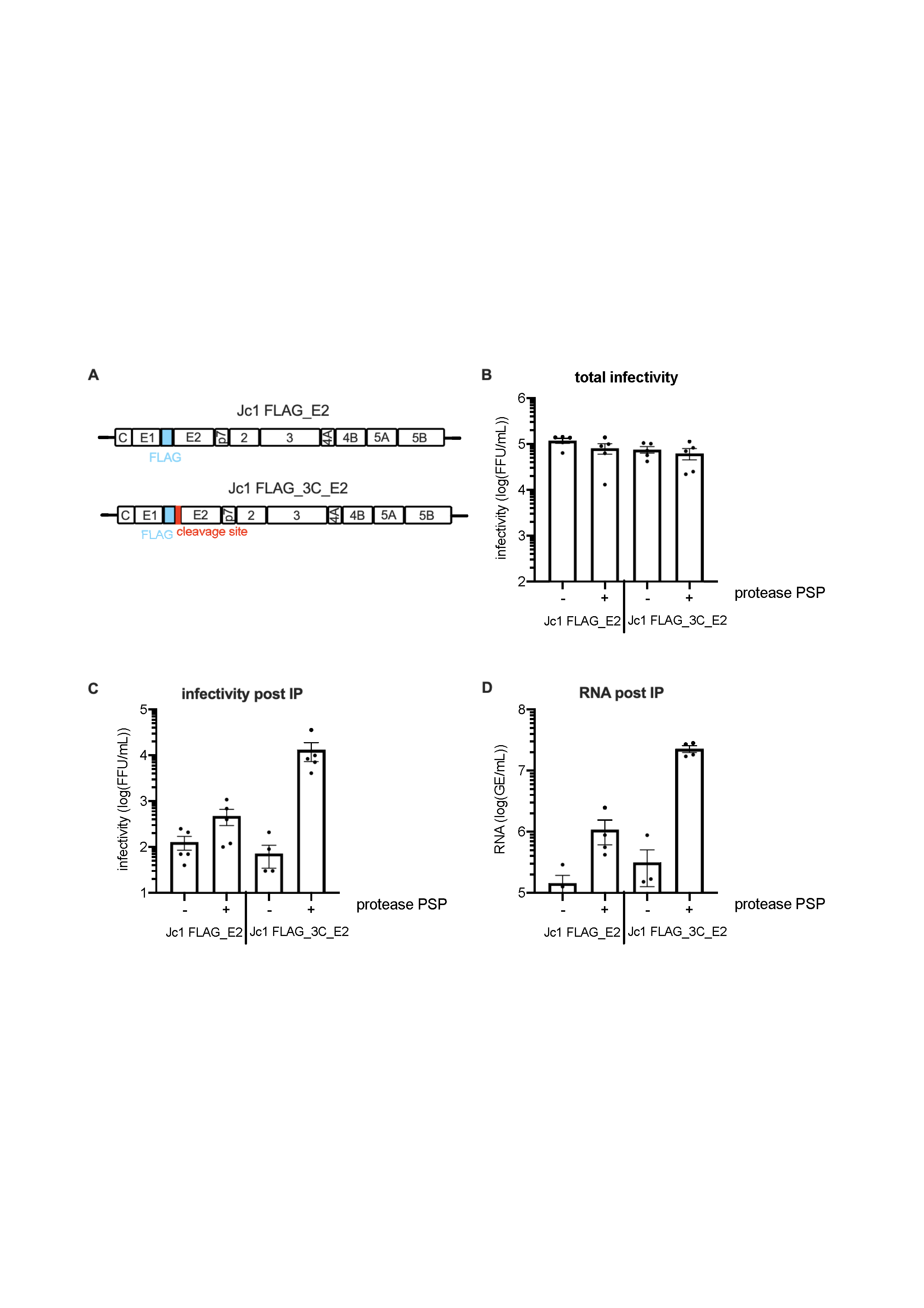

Supplement: Fig. S1 — Addition of a cleavage site between FLAG and E2 sequences. [file mbio.01549-23-s0001.tif]

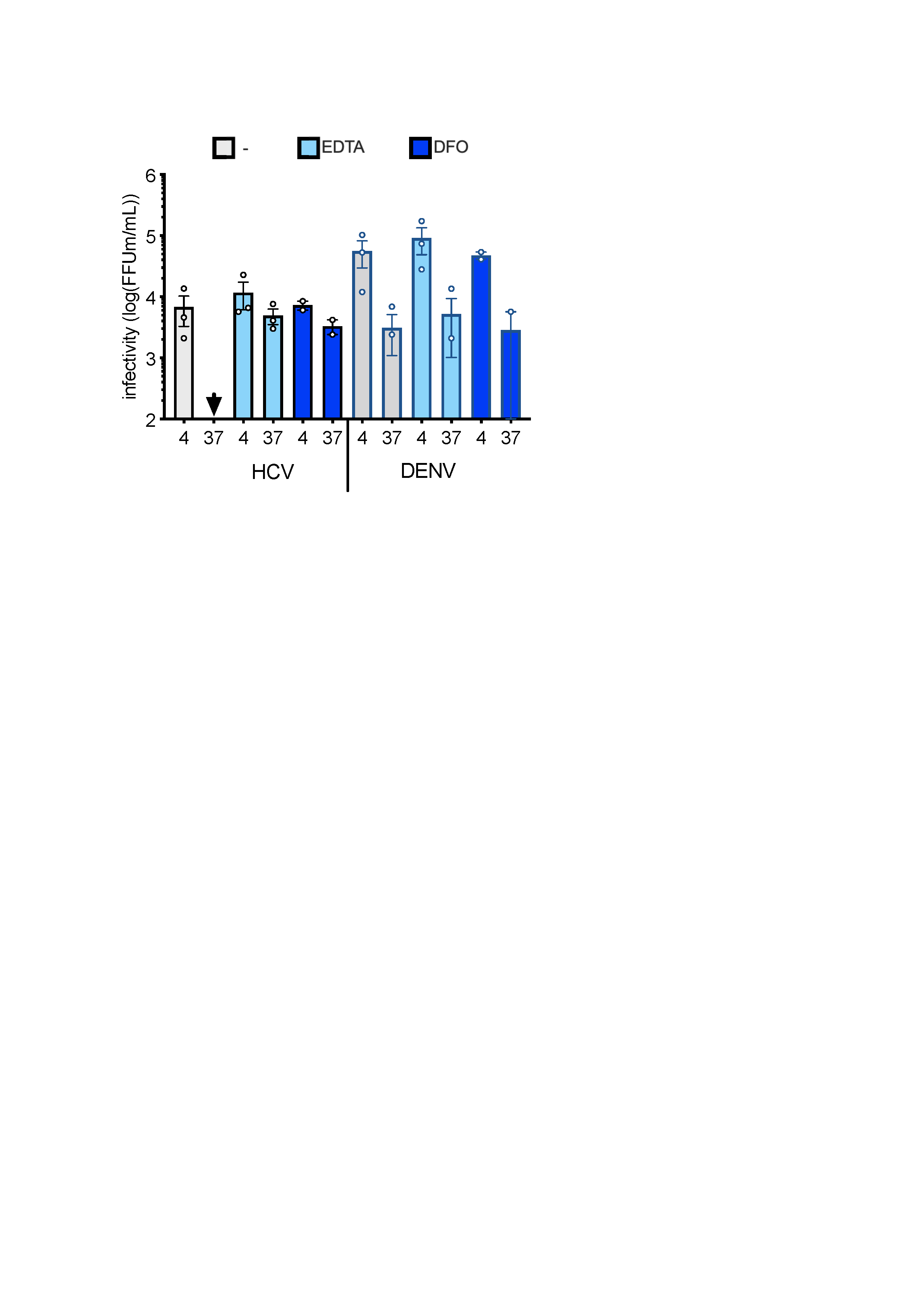

Supplement: Fig. S2 — No effect of DFO and EDTA on intracellular DENV particles. [file mbio.01549-23-s0002.tif]

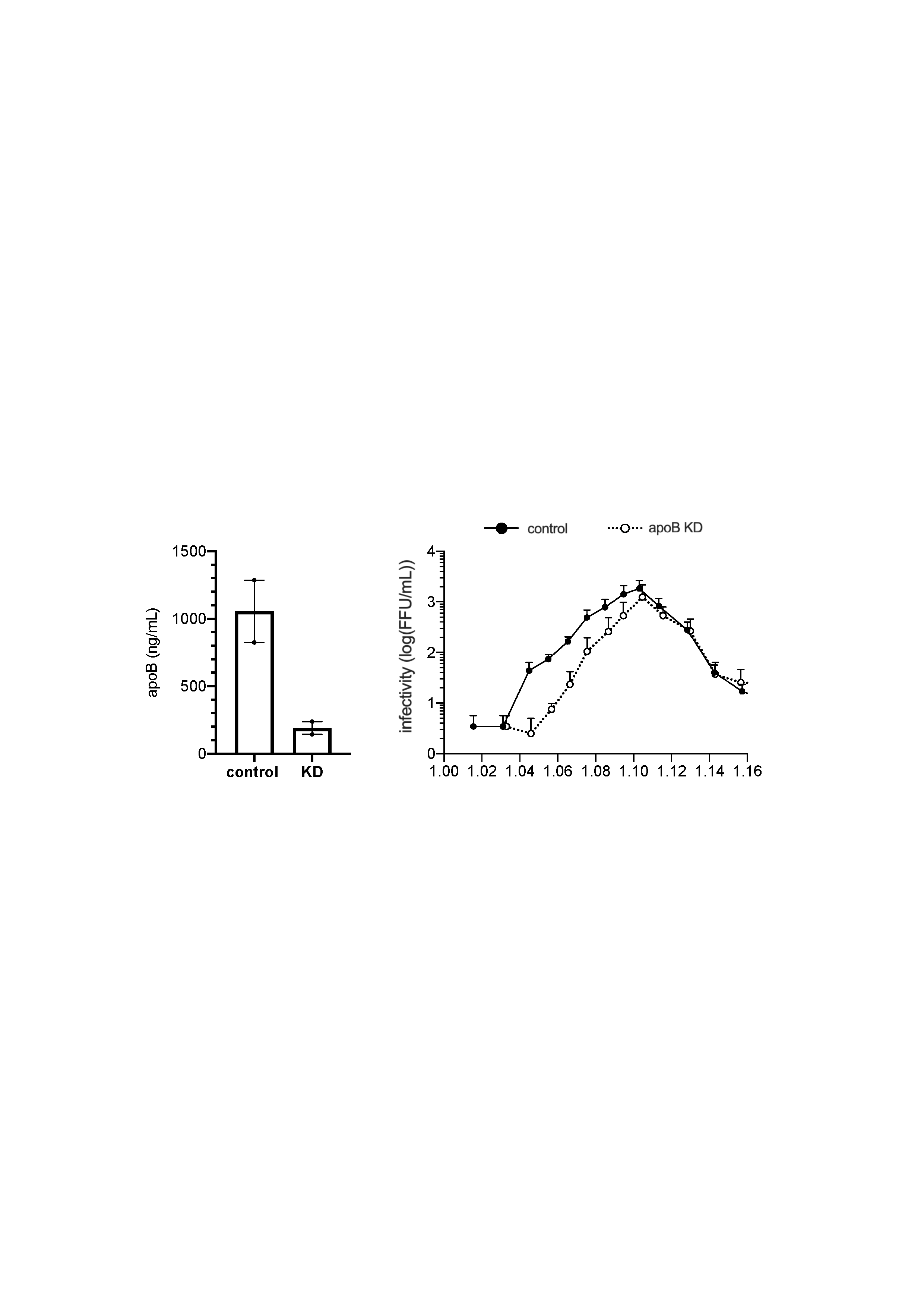

Supplement: Fig. S3 — Effect of knock-down of apoB on density gradient of HCVcc. [file mbio.01549-23-s0003.tif]
