# Supplementary material for: Low-density hepatitis C virus infectious particles are protected from oxidation by secreted cellular proteins
Source: mBio. 2023 Sep 6;14(5):e01549-23. doi: 10.1128/mbio.01549-23 (PMC10653866; doi:10.1128/mbio.01549-23)
Supplement: Supplemental Legends — Supplemental figure legends. [file mbio.01549-23-s0004.docx]

**Supplemental Figure legends**

**Low density hepatitis C virus infectious particles are protected from oxidation by secreted cellular proteins**

**Running title**

Co-secreted cellular factors protect HCV from oxidation

Christelle Granier^1#^, Johan Toesca^1#^, Chloé Mialon^1#^, Maureen Ritter^1^, Natalia Freitas^1^, Bertrand Boson^1^, Eve-Isabelle Pécheur^2^, François-Loïc Cosset^1$^, Solène Denolly^1,3 $,*^

**Affiliations**

^1^CIRI – Centre International de Recherche en Infectiologie, Univ. Lyon, Université Claude Bernard Lyon 1, Inserm, U1111, CNRS, UMR5308 ENS de Lyon, F-69007, Lyon, France.

^2^Univ Lyon, Université Claude Bernard Lyon 1, CNRS 5286, INSERM 1052, Centre Léon Bérard, Centre de Recherche en Cancérologie de Lyon, 69008, Lyon, France.

^3^Department of Infectious Diseases, Molecular Virology, Heidelberg University, 69120 Heidelberg, Germany.

^#^co-first authors

^$^co-last authors

* Corresponding author

**Supplemental Figure 1. Addition of a cleavage site between FLAG and E2 sequences**.

**(A)** Representation of the modified polyproteins. **(B)** Infectivity of crude supernatant treated or not with PreScission Protease (PSP). **(C)** Infectivity of Jc1 FLAG_E2 or Jc1 FLAG_3C_E2 recovered after IP anti FLAG and elution using cleavage of the FLAG tag with PSP. **(D)** RNA of Jc1 FLAG_E2 or Jc1 FLAG_3C_E2 recovered after IP anti FLAG and elution using cleavage of the FLAG tag with PSP.

The results are represented as means ± SEM. Each dot in the graphs corresponds to the value of an individual experiment.

**Supplemental Figure 2. No effect of DFO and EDTA on intracellular DENV particles.** Infectivity of HCV and DENV intracellular particles diluted in serum-free medium (-) *vs*. in serum-free medium supplemented with EDTA (0.5mM) or DFO (10μM).

The results are represented as means ± SEM. Each dot in the graphs corresponds to the value of an individual experiment.

**Supplemental Figure 3. Effect of knock-down of apoB on density gradient of HCVcc**

**(A)** Level of secreted apoB by Huh7.5 (control) and Huh7.5 transduced by shRNA targeting apoB (KD). **(B)** Infectivity of HCV particles detected in fractions from buoyant density gradients of crude virus producer cell supernatant from either Huh7.5 (full line) and Huh7.5 with knock-down of apoB (dotted line).

The results are represented as means ± SEM.
